# Tree species diversity promotes soil microbial carbon fixation gene abundance via nutrient-mediated interactions in subtropical forests

**DOI:** 10.3389/fmicb.2026.1751295

**Published:** 2026-02-10

**Authors:** Siwen Su, Jinwen Pan, Huili Wu, Shuai Ouyang, Liang Chen, Yelin Zeng, Nan Deng

**Affiliations:** 1 College of Ecology and Environment, Central South University of Forestry and Technology, Changsha, Hunan, China; 2 Huitong National Station for Scientific Observation and Research of Chinese Fir Plantation Ecosystems in Hunan Province, Huitong, Hunan, China; 3 Hunan Academy of Forestry, Changsha, Hunan, China; 4 Hunan Cili Forest Ecosystem State Research Station, Cili, Hunan, China

**Keywords:** gene abundance, microbial carbon fixation, soil properties, subtropical forest, tree species diversity

## Abstract

Soil microbial carbon (C) fixation represents a vital yet uncertain component of forest carbon cycling, and its underlying mechanisms especially depth-specific responses remain unclear. To address this, we integrated metagenomics and machine learning to examine these relationships along a tree species richness gradient (1–8 species), analyzing both topsoil (0–10 cm) and subsoil (10–20 cm). Results revealed distinct vertical stratification in soil properties and microbial carbon fixation strategies. Microbial carbon fixation gene abundance was primarily driven by soil organic carbon (SOC) and nitrate nitrogen (NO₃^−^-N), exhibiting a nonlinear threshold at ~85 g kg^−1^ SOC. The promoting effect of SOC peaked at moderate richness (3–5 species) but declined at higher richness. Depth-resolved analysis revealed that the Calvin cycle gene *rbcL* responded mainly to richness in topsoil, whereas rTCA cycle genes (*korA, korC*) were more sensitive in subsoil These findings demonstrate that tree diversity enhances microbial carbon fixation through nutrient-mediated mechanisms, but these effects are nonlinear, context-dependent, and depth-specific. Incorporating such complexity is essential for accurately predicting forest carbon sequestration.

## 1 Introduction

Forest ecosystems are major regulators of the global carbon cycle, storing approximately 226 Gt of carbon (Zhu et al., 2021). In contrast to the well-studied aboveground biomass accumulation (Yang et al., 2023), the belowground carbon cycle-driven by intricate interactions between plant inputs, soil properties, and microbial metabolism-remains poorly understood (Wu et al., 2024). Tree species diversity has been shown to enhance ecosystem carbon storage (Liang et al., 2016), but the mechanistic pathways linking aboveground diversity to belowground C fixation processes are not well resolved.

The aboveground-belowground linkage operates primarily through two pathways: (1) litter inputs, which provide organic carbon substrates for heterotrophic decomposition, and (2) root exudates, which fuel microbial metabolism in the rhizosphere (Bardgett and van der Putten, 2014). Tree species diversity can amplify these inputs through niche complementarity, where chemically distinct litter and exudates from diverse species create heterogeneous resource environments that support specialized microbial functional guilds (Sasse et al., 2018). However, the extent to which this plant-derived carbon is channeled into microbial autotrophic C fixation-a process that directly converts atmospheric CO₂ into soil organic carbon (SOC)-remains unclear.

Soil microorganisms fix CO₂ via six principal autotrophic pathways: the Calvin cycle, the reductive tricarboxylic acid cycle (rTCA), the 3-hydroxypropionate bi-cycle (3-HP), the 3-hydroxypropionate/4-hydroxybutyrate cycle (3-HP/4-HB), the dicarboxylate/4-hydroxybutyrate cycle (DC/4-HB), and the Wood–Ljungdahl (WL) pathway (Table 1). The abundance and activity of pathway-associated genes are jointly regulated by multiple environmental factors, including SOC, nitrogen and phosphorus availability, pH, and moisture (Li et al., 2023; Luo et al., 2020; Malik et al., 2018; Naz et al., 2022). Critically, these drivers do not act in isolation; their effects are often contingent on nutrient stoichiometry and energetic trade-offs (Liang et al., 2024). For instance, the Calvin cycle, while kinetically robust, is energetically expensive (requiring 7 ATP and 4 NADPH per CO_2_), making it favorable only in resource-replete, oxygen-rich environments. In contrast, the energy-efficient rTCA cycle (requiring 2 ATP) provides a competitive advantage in resource-limited or microaerophilic niches (Bar-Even et al., 2012; Berg, 2011).

**Table 1:** Six main metabolism pathways of soil microbial C fixation.

C fixation pathways	Key enzymes	Function genes	Reference
Calvin cycle	Ribulose-1,5-bisphosphate carboxylase (RubisCO)	*rbcL(cbbL)*	Foyer and Halliwell (1976) and Stincone et al. (2015)
rTCA cycle	Pyruvate: Ferredoxin oxidoreductase; 2-oxoglutarate synthase; Succinyl-CoA: citrate synthase	*korA, korB, korC, korD, por*	Akinyede et al. (2020), Campbell and Cary (2004) and Liu et al. (2018)
4-HB cycle	Malonyl-CoA reductase; Phosphoenolpyruvate carboxylase; 4-Hydroxybutyryl-CoA dehydratase	*hcd*	Huber et al. (2008)
3-HP/4-HB cycle	Acetyl-CoA/propionyl-CoA carboxylase; 4-Hydroxybutyryl-CoA dehydratase	*atoB*	Berg et al. (2007)
3-HP cycle	Malonyl-CoA reductase; Malyl-CoA/β-methylmalyl-CoA/citramalyl-CoA lyase	*atoB, ppcA*	Shih et al. (2017)
Wood–Ljungdahl pathway	Acetyl-CoA synthase; CO dehydrogenase	*acsB, fhs, acsA, hyc*	Berg (2011)

Microbial C fixation pathways, such as the Calvin cycle and the reductive tricarboxylic acid (rTCA) cycle, are energetically costly but ecologically significant contributors to soil carbon sequestration (Berg et al., 2010; Hügler and Sievert, 2011). These pathways are regulated by the interplay between biotic factors (e.g., microbial community composition, plant diversity) and abiotic factors (e.g., soil pH, nutrient availability, redox conditions). For instance, the Calvin cycle, which requires high ATP and NADPH inputs, is typically favored in oxygen-rich, nutrient-replete topsoil, whereas the energy-efficient rTCA cycle may dominate in resource-limited, microaerophilic subsoil (Bar-Even et al., 2012). Yet, how vertical gradients in soil physicochemical properties-shaped by long-term litter accumulation, root distribution, and biogeochemical turnover-structure the spatial distribution of these pathways remains poorly quantified. Despite these theoretical underpinnings, the vertical stratification of forest soils creates contrasting microenvironments that challenge linear assumptions of microbial function. Topsoil typically receives concentrated litter inputs and maintains higher oxygen availability, whereas subsoils are characterized by steep declines in redox potential and nutrient accessibility (Keiluweit et al., 2020; Zhang and Furman, 2021). While these vertical gradients likely drive depth-specific adaptations in carbon-fixation strategies, empirical evidence remains scarce. Furthermore, tree diversity effects on these processes may be nonlinear and context-dependent, potentially exhibiting unquantified thresholds where high stand density or resource competition suppresses microbial activity (Standish et al., 2022; Wang et al., 2021).

To bridge these gaps, frameworks that can capture nonlinearity and multidimensional interactions are urgently needed. Traditional linear models often oversimplify the complex above-belowground linkages by treating soils as homogeneous compartments. Gradient boosting machine (GBM) models offer a powerful alternative to identify non-obvious interactions and tipping points among multiple predictors (Ma et al., 2020). While widely applied in aquatic systems, their potential to unravel the complexities of forest soil C fixation remains largely untapped.

In this study, we conducted a metagenomic investigation in the Dashanchong permanent forest plots in subtropical China-a biodiversity hotspot characterized by high structural complexity and species richness (Ehbrecht et al., 2021). We established a tree-species richness gradient (1–8 species per subplot across 36 subplots) and explicitly distinguished topsoil (0–10 cm) and subsoil (10–20 cm) to capture vertical heterogeneity. Combining GBM with multiple regression tree (MRT) analyses, we aimed to: (1) quantify nonlinear relationships and thresholds linking tree-species richness to carbon-fixation gene abundance; (2) identify interaction effects between the soil environment and tree diversity on carbon-fixation genes and their thresholds; and (3) elucidate layer-specific mechanisms by which tree diversity influences key functional genes in major pathways. We hypothesized that: (i) diversity effects on gene abundance would show threshold-type rather than purely linear responses; (ii) interactions between the soil environment and tree diversity significantly affect gene abundance and produce nonlinear responses; and (iii) topsoil versus subsoil would exhibit distinct gene–diversity relationships, reflecting depth-dependent shifts in dominant carbon-fixation pathways. Our study provides mechanistic insights into how plant diversity shapes belowground carbon cycling and offers actionable guidance for forest management aimed at maximizing carbon sequestration through biodiversity conservation.

## 2 Materials and methods

### 2.1 Site description and experimental design

This study was conducted in Dashanchong Forest Park (28°23′58″–28°24′58″N, 113°17′46″–113°19′08″E), located in Changsha County, Hunan Province, China (Supplementary Figure S1). The region is characterized by a humid subtropical monsoonal climate, with an average annual temperature of 17.3 °C and mean annual precipitation of 1,416 mm (Liu et al., 2014). The soil is a well-drained clay loam, classified as Alliti-Udic Ferrosol (WRB, IUSS et al., 2015). Secondary forests dominate the park, with human disturbance strictly prohibited since the late 1950s following the implementation of forest protection policies in China.

Two 1.0-hectare permanent plots were established, each plot divided into 10 m × 10 m subplots. Forest resources were surveyed every 5 years, with the most recent survey conducted in 2023 (Supplementary Table S1). To investigate relationships between tree species diversity, soil properties, and C fixation gene abundance across soil layers, subplots were selected based on survey data and the following criteria: (1) maximizing the range of tree species richness to ensure representativeness and reduce outliers; (2) selecting three or more replicates per richness level to ensure statistical reliability; (3) maintaining a minimum 10-meter distance between selected subplots to minimize spatial autocorrelation,; and (4) ensuring consistency of other geo-climatic conditions among subplots. Following these criteria, 36 subplots with tree species richness ranging from 1 to 8 were selected for subsequent analyses (Supplementary Table S2). This 10-meter interval was strategically chosen based on the stand structure of the Dashanchong forest, where the typical crown radius and lateral root extension of dominant trees range from 2 to 5 m. By maintaining a 10 m buffer, we ensured that the soil samples from each subplot primarily reflected the biological influence of the target tree community. Furthermore, the study site is characterized by high canopy closure (average >85%), which significantly limits light penetration to the forest floor. Consequently, the understory herb layer is extremely sparse and exerts negligible influence on the soil carbon cycle in this system. This allows for a more precise evaluation of how tree species richness—rather than understory vegetation—shapes the microbial carbon-fixation potential through litter and root-derived inputs. Tree density and tree species diversity indices (richness and Shannon index) were calculated for each subplot.

### 2.2 Soil sampling and physicochemical property analysis

Soil samples were collected from the 36 subplots. For each subplot, seven random soil cores were homogenized by layer (topsoil: 0-10 cm; subsoil: 10–20 cm) using a soil auger (10 cm diameter, 20 cm depth) to form composite samples, resulting 72 soil samples (36 subplots × 2 layers). Samples were immediately sieved through a 2 mm mesh to remove stones and plant debris. One portion was stored at −80 °C for DNA extraction and metagenomic sequencing, another portion was stored at 4 °C for analysis of soil moisture, microbial biomass carbon (MBC), microbial biomass nitrogen (MBN), microbial biomass phosphorus (MBP), available phosphorus (AP), and inorganic nitrogen (NH_4_^+^-N and NO₃^−^-N). Remaining samples were air-dried for analysis of soil organic carbon (SOC), total phosphorus (TP), total nitrogen (TN), and pH.

Soil pH was measured at a soil-to-water ratio of 1:2.5 using a pH meter (Mettler Toledo Instrument Co., Ltd., Shanghai, China). Soil moisture content was determined gravimetrically after drying at 105 °C to constant weight. SOC was quantified using the K_2_Cr_2_O_7_-H_2_SO_4_ oxidation method. TP was measured via the Mo-Sb colorimetric method, and TN was assessed using the Kjeldahl method (Hao et al., 2024). Soil inorganic nitrogen was extracted with 0.5 M K_2_SO_4_ solution, and the concentrations of NH_4_^+^-N and NO₃^−^-N in the filtered extract were measured using a flow injection analyzer (FIAstar 5,000, FOSS, Höganäs, Sweden). MBC, MBN, and MBP were determined using the chloroform fumigation-extraction method, with a conversion coefficient of 0.45 (Zhang et al., 2013). AP was determined using a solution of 0.05 M HCl and 0.025 M H_2_SO_4_ (Mehlich, 1984).

### 2.3 DNA extraction and metagenomics analysis

Total genomic DNA was extracted from 0.25 g soil using the E. Z. N. A.^®^ Soil DNA Kit (Omega Bio-tek, Norcross, GA, United States). Metagenomic libraries were constructed with fragment lengths of approximately 400 bp using Covaris M220 (Gene Company Limited, Shanghai, China) for size selection and NEXTFLEX^®^ Rapid DNA-Seq (Bio Scientific, Austin, TX, USA) for library preparation. The size-selected libraries were sequenced on the Illumina NovaSeq platform (Illumina Inc., San Diego, CA, United States) in paired-end mode (2 × 150 bp).

Raw reads were quality-filtered using fastp software (version 0.20.0) (Chen et al., 2018) on the Majorbio Cloud Platform^1^. Adaptor sequences were trimmed, and reads shorter than 50 bp, with quality values below 20, or containing N bases were removed. High-quality reads were assembled into contigs using MEGAHIT (Li et al., 2017), and contigs ≥300 bp in length were retained. Open reading frames (ORFs) were predicted using MetaGene (Noguchi et al., 2006), and genes with nucleotide lengths longer than 100 bp were selected and translated into amino acid sequences. A non-redundant gene catalog was constructed using CD-HIT (Fu et al., 2012) with 95% sequence identity and 90% coverage thresholds. Assembly quality was assessed by calculating the N50 statistic (12.5 kbp) and completeness using BUSCO (v5.4.4) against the bacteria_odb10 lineage dataset, yielding 92.7% completeness. High-quality reads were mapped to the non-redundant gene catalog using SOAPaligner (Li et al., 2008), and gene abundance was quantified for each sample.

Representative sequences from the non-redundant gene catalog were taxonomically annotated by alignment against the NCBI NR database using Diamond software (Buchfink et al., 2015) with an e-value cutoff of 1 × 10^−5^. Functional annotation was performed against the Kyoto Encyclopedia of Genes and Genomes (KEGG) database using the same e-value threshold. The abundance of each taxonomic group was calculated by summing the abundance of genes annotated to that group. Relative gene abundances (%) were normalized across all samples based on annotated read counts. Gene abundances were normalized across samples using variance stabilizing transformation in the DESeq2 R package (Love et al., 2014) to account for differences in sequencing depth. Relative abundances were then calculated as the proportion of each gene’s normalized count to the total normalized counts of all annotated genes within each sample. Subsequent analyses focused on genes involved in C fixation pathways.

### 2.4 Validation of key gene abundances via qPCR

Quantitative PCR was performed for *rbcL* (Calvin cycle) and *korA* (rTCA cycle) to validate metagenomic abundance estimates (Supplementary Table S3). Standard curves were generated using serial dilutions of plasmid DNA containing target gene fragments. Gene copy numbers per gram of dry soil were calculated. qPCR derived abundances strongly correlated with metagenomic read counts for both genes (Supplementary Figure S3), confirming the reliability of sequencing-based quantification.

### 2.5 Statistical analysis

All statistical analyses were performed in R software (version 4.2.2; R Development Core Team, 2022). Differences in soil physicochemical properties between soil layers were assessed using one-way analysis of variance (ANOVA), with statistical significance set at *p* < 0.05. Violin plots were generated to visualize the distribution and variability of soil properties across the two layers. Variations in C fixation gene composition were assessed using analysis of similarity (ANOSIM) with the “*vegan*” R package and visualized using nonmetric multidimensional scaling (NMDS) based on Bray–Curtis dissimilarity matrix. Permutational multivariate analysis of variance (PERMANOVA, permutations = 999) was performed to test for statistical significance of group differences. Heatmaps with hierarchical clustering visualized gene expression patterns across pathways and soil layers. To identify key environmental drivers of gene abundance, gradient boosting machine (GBM) models were fitted using the “*gbm*” package (Chen et al., 2011). GBM was selected for its ability to capture complex nonlinear relationships and interactions between predictor variables in ecological gradient data (Ma et al., 2020). Relative importance of variables was assessed, and partial dependence plots were generated to visualize the marginal effects of key factors on gene abundance. Two-way partial dependence plots were constructed to examine interactive effects between SOC concentration and tree species richness. Model performance was evaluated using 10-fold cross-validation, and the optimal number of trees was selected to minimize the cross-validated error.

Generalized Additive Models (GAM) examined nonlinear relationships between tree species richness and NMDS ordination scores. Spearman correlation analysis assessed relationships between environmental factors and gene abundance across pathways. Linear regression analyses were conducted to examine relationships between tree species richness and the abundance of key functional genes in C fixation pathways (*rbcL, korA, korC, korD, por*) separately for topsoil and subsoil layers. Multiple regression tree (MRT) analysis (De’ath, 2001) was performed using the “*mvpart*” package to identify threshold values of environmental variables that best explained variation in C fixation gene composition. The MRT model partitioned samples into subgroups based on soil and stand factors, and indicator species analysis was conducted to identify genes characteristic of each subgroup (indicator value, *p* < 0.05). Model performance was evaluated using error, cross-validation error, and standard error metrics.

## 3 Results

### 3.1 Soil physicochemical properties

**Figure 1:**
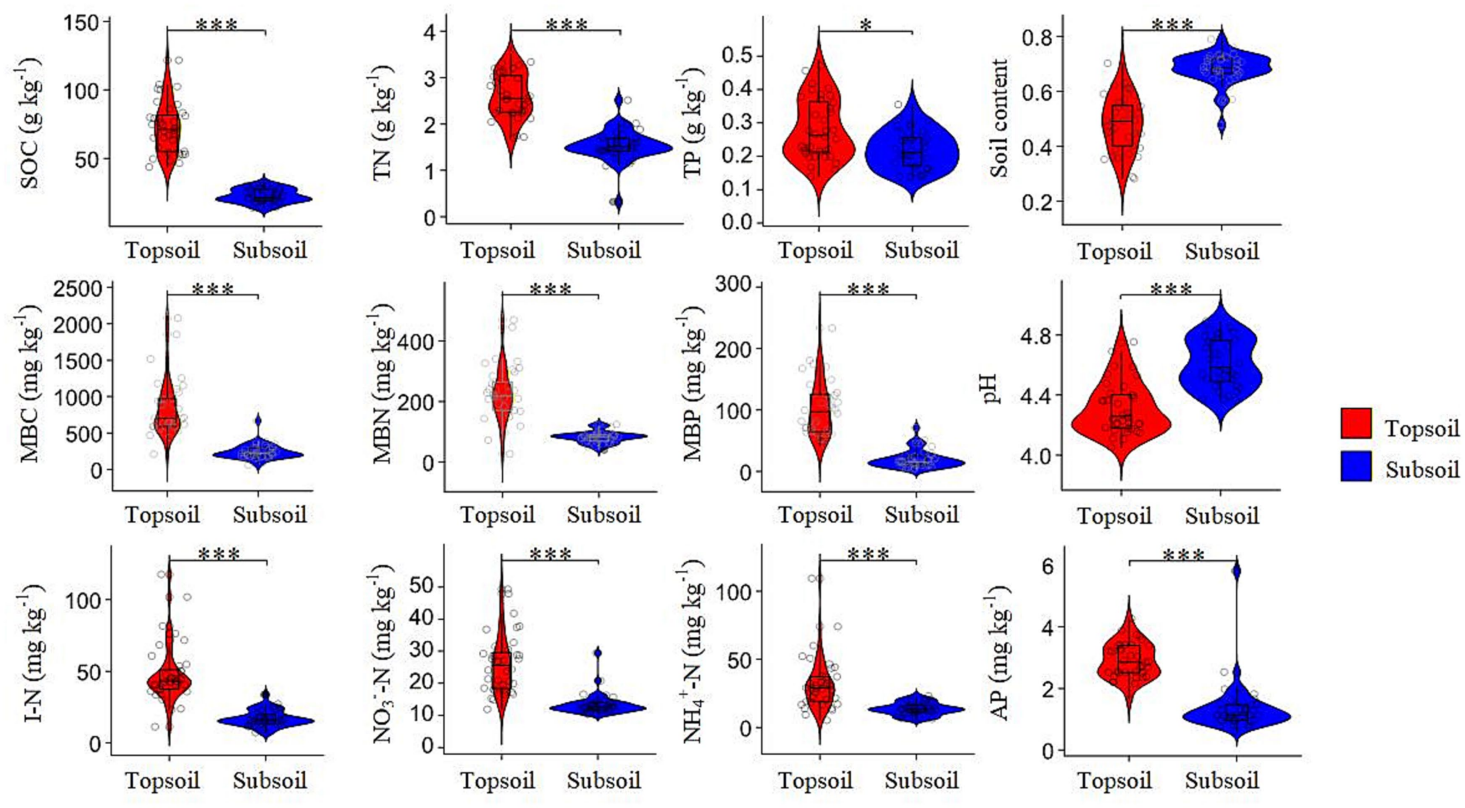
Comparison of the physical and chemical properties between topsoil and subsoil. SOC: Concentration of soil organic carbon (g kg^−1^); TN: Concentration of soil total nitrogen (g kg^−1^); TP: Concentration of soil total phosphorus (g kg^−1^); MBC: Microbial biomass carbon (mg kg^−1^); MBN: Microbial biomass nitrogen (mg kg^−1^); MBP: Microbial biomass phosphorus (mg kg^−1^); I-N: Total soil inorganic nitrogen (mg kg^−1^); NO_3_^−^-N: Soil nitrate nitrogen (mg kg^−1^); NH_4_^+^-N: Soil ammonium nitrogen (mg kg^−1^); AP: Soil available phosphorus (mg kg^−1^). The significance levels of each predictor are *p < 0.05, **p < 0.01, ***p < 0.001.

Soil physicochemical properties exhibited significant differences between topsoil and subsoil (Figure 1). Violin plots showed distinct distribution patterns, with topsoil generally exhibiting narrower distributions. Compared to topsoil, subsoil had significantly higher pH but lower values for all other measured parameters (*p* < 0.05), including SOC, TN, TP, MBC, MBN, MBP, I-N (total soil inorganic nitrogen), NO_3_^—^N, NH_4_^+^-N. These vertical distribution patterns reflect clear stratification of soil properties in the forest ecosystem.

### 3.2 Patterns of C fixation gene abundance and pathways

**Figure 2:**
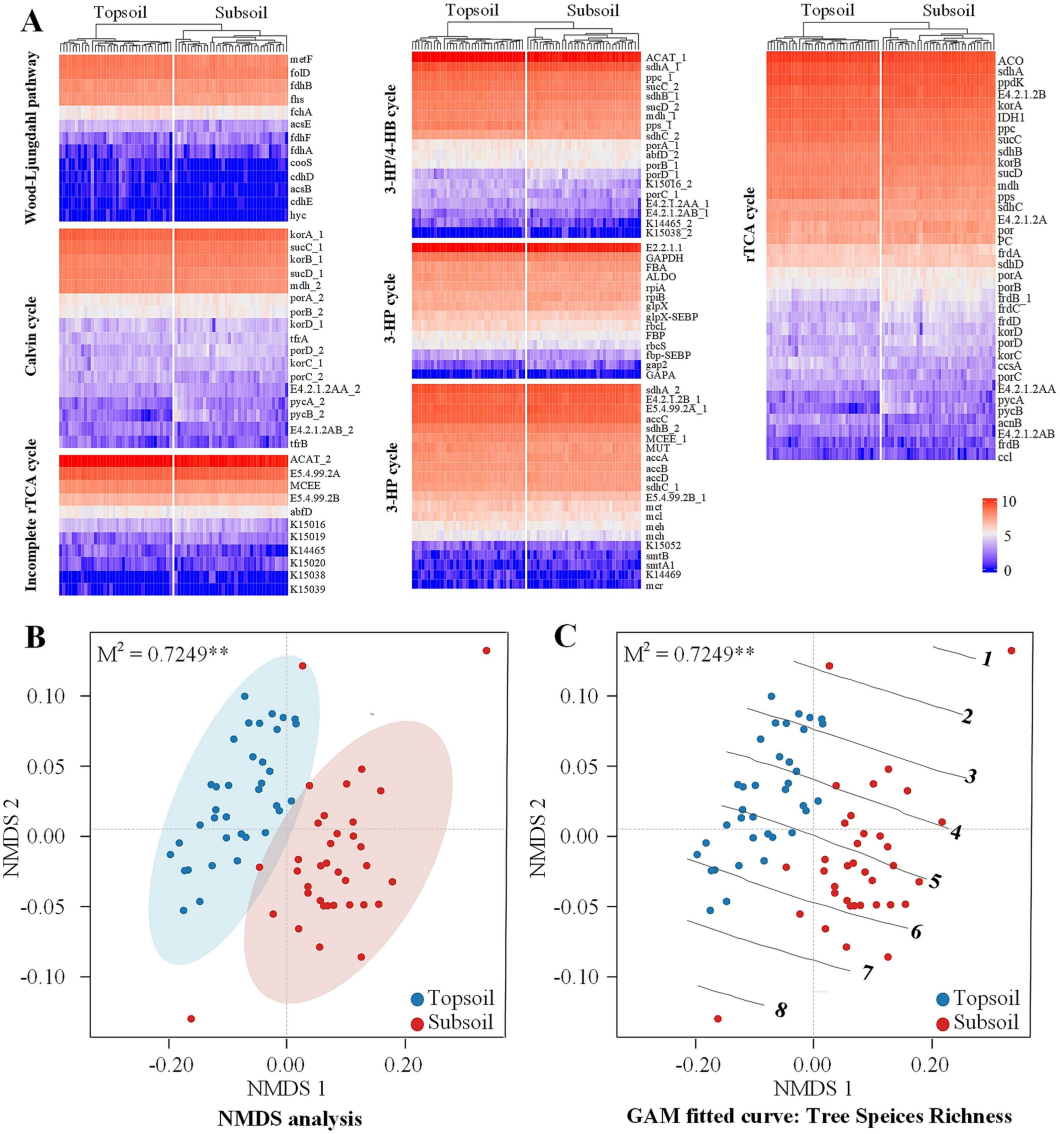
Differential response of C fixation-related gene abundances to soil conditions and tree species diversity indices. **(A)** Heatmap and cluster analysis of main gene abundance for 7 main cycles (pathways) of C fixation in different soil layers. **(B)** Procrustes analysis of the correlation between gene abundance and soil conditions with 999 permutations based on NMDS using Bray–Curtis distance (*M*^2^ = 0.7249, *p* = 0.001). **(C)** Generalized additive model (GAM) fitted curves of tree species richness (gradients of 1–8), the numbers on the curve represent the model arithmetic fitting values to the trend surface. The residuals for each set of paired values in the Procrustes analysis are shown in Supplementary Figure S2.

Heatmap clustering revealed similar gene profiles across seven C fixation metabolic pathways between soil layers (Figure 2A). Highly abundant genes were identified in the rTCA cycle, the Calvin cycle, and the 3-HP cycle. Specifically, genes in the rTCA cycle (*korA, ACO, sdhA, ppdK*, and *sucC*), Calvin cycle (*rbcL*, *E2.2.1.1*), 3-HP cycle (*sdhA 2, E4.2.1.2B, E5.4.99.2A*, *accC*), and 3-HP/4-HB cycle (*ACAT*) exhibited higher relative abundance. Two genes in the Wood–Ljungdahl pathway (*metF*, *folD*) also showed higher abundance. Genes with lower abundance were found in the rTCA cycle (*frdB*, *ccl*), 3-HP cycle (*K14469*, *mcr*), Calvin cycle (*GAPA*), 4-HB cycle (*K15038*), 3-HP/4-HB cycle (*K15038*, *K15039*), and Wood-Ljungdahl pathway (*hyc*).

The NMDS indicated significant separation of C fixation-related genes between soil layers (*M*^2^ = 0.7249, *p* = 0.001, Figure 2B). Fitting curves of tree species richness displayed a linear, nearly parallel trend between soil layers, with samples distributed along these gradients (Figure 2C), suggesting that the composition of C fixation genes varied with tree species richness.

### 3.3 Key environmental factors driving C fixation gene abundance

**Figure 3:**
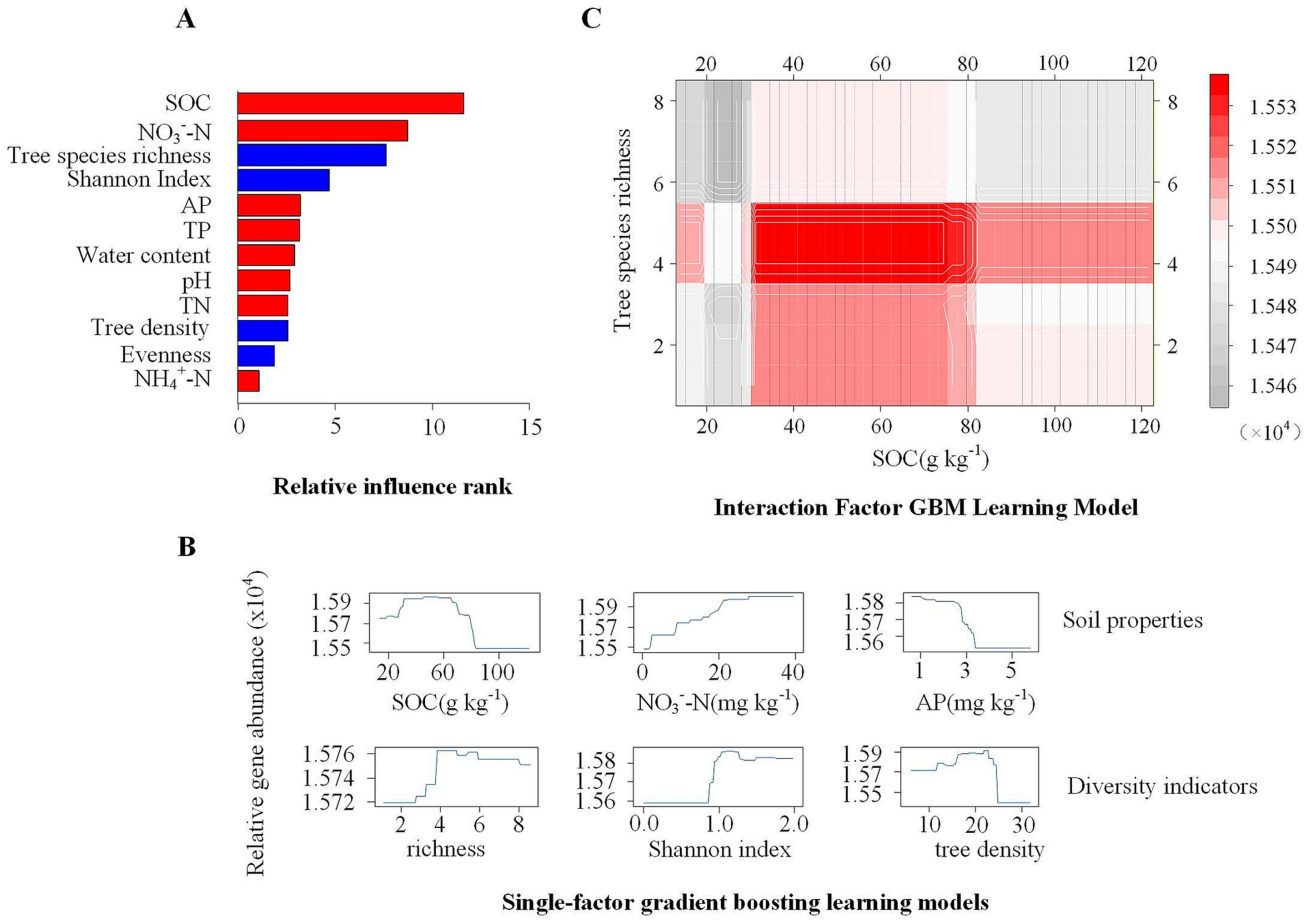
Modeling the effect of environmental factors on the total abundance of C fixation-related genes using a gradient boosting machine. **(A)** Influence importance ranking of drivers in 2 categories on gene abundance; blue represents tree diversity indices, and red represents soil properties. **(B)** Univariate fit relationships between the top 3 drivers of importance in different categories and gene abundance. **(C)** Modeling the interaction effect of tree species richness and soil organic carbon (SOC) on the abundance of C fixation-related genes. The color from gray to red indicates an increase in gene abundance.

The GBM model was employed to rank the influence of soil properties on the abundance of C fixation-related genes (Figure 3A). SOC and NO₃^−^-N concentrations were identified as the most influential factors affecting C fixation gene abundance, followed by tree species richness and Shannon index. Partial dependence plots revealed distinct response patterns: gene abundance gene abundance initially increased with rising SOC concentration, peaked, then decreased, and stabilized beyond a threshold of 84.72 g kg^−1^ (Figure 3B). In contrast, NO₃^−^-N and AP content exhibited opposite trends: gene abundance increased with NO₃^−^-N content, whereas it decreased as AP content rose. As tree species diversity increased, gene abundance initially increased before leveling off, indicating a saturating diversity effect.

**Figure 4:**
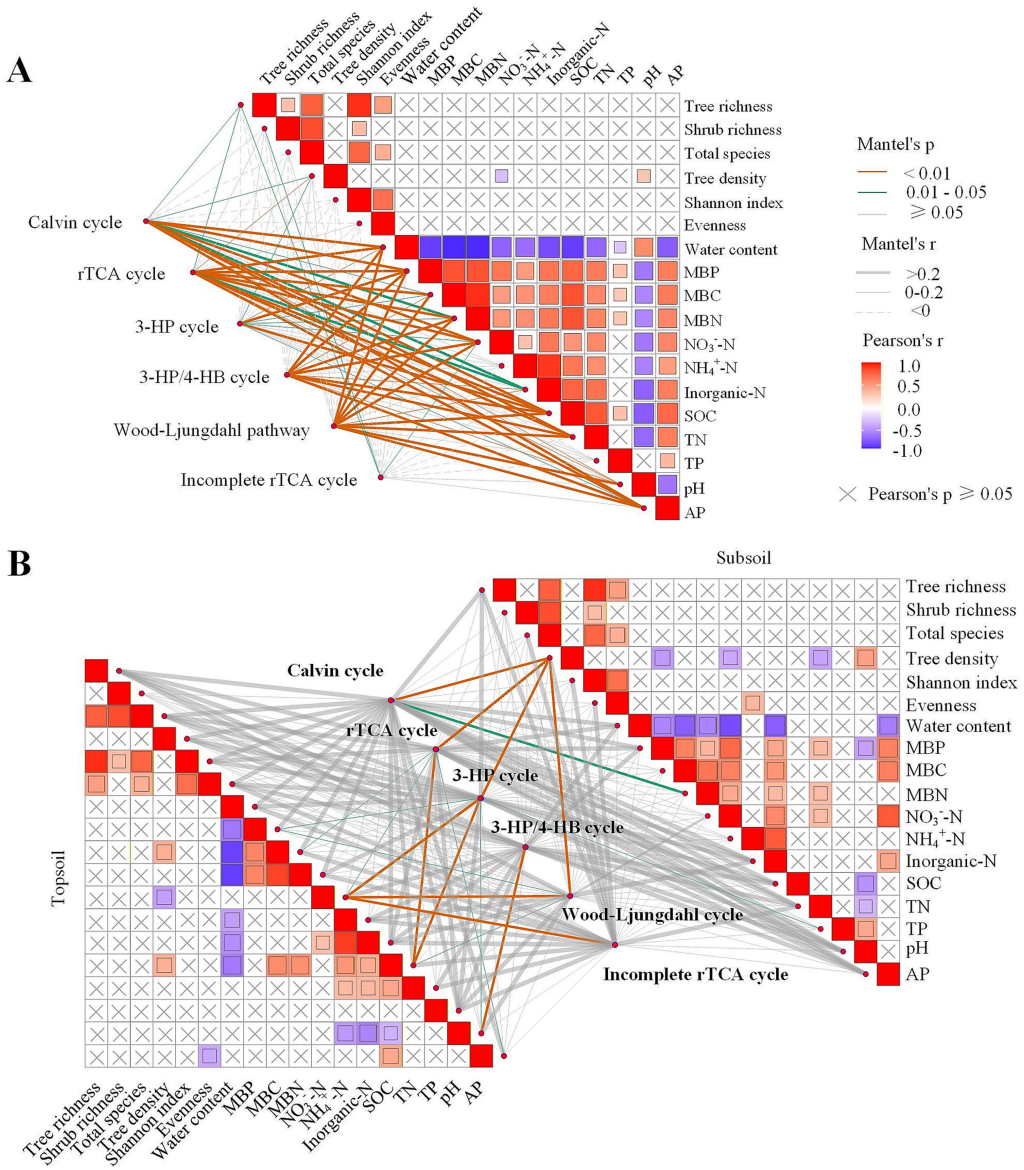
Pairwise comparisons of environmental factors are shown with the color gradient denoting Spearman’s correlation coefficients. Cycles (pathways) of C fixation were related to each environmental factor by partial (geographic distance-corrected) Mantel tests. Edge width corresponds to Mantel’s r statistic for the corresponding distance correlations, and edge color denotes the statistical significance based on 9,999 permutations. Results with no significant correlation (*p* > 0.05) in mental testing were not shown briefly. **(A)** Shows the results for all samples, while **(B)** Presents results for topsoil (lower-left triangle) and subsoil (upper-right triangle).

Analysis of marginal effects revealed interactive influences between SOC and tree species richness on gene abundance (Figure 3C). At moderate levels (approximately 3 to 5), SOC had the strongest effect, promoting gene abundance up to peak near 80 g kg^−1^. However, as tree species richness increased, SOC’s influence gradually weakened. Particularly, stabilizing at higher levels of tree species richness, suggesting that tree species diversity imposes a saturating effect on SOC influence. Correlation analysis revealed distinct associations between soil environmental factors and different C fixation metabolic pathways (Figure 4A). Across all samples, the Calvin cycle showed significant correlations with the broadest spectrum of environmental variables, including tree richness, tree density, water content, MBP, MBN, NO₃^−^-N, inorganic-N, SOC, TN, AP, and pH. The rTCA cycle was significantly correlated with water content, MBP, MBN, NO₃^−^-N, inorganic-N, SOC, TN, AP, and pH. The 3-HP cycle exhibited significant correlations with tree richness, water content, MBP, MBN, NO₃^−^-N, and AP. The 3-HP/4-HB cycle was significantly correlated with water content, MBP, MBN, NO₃^−^-N, inorganic-N, SOC, TN, AP, and pH. The Wood–Ljungdahl pathway showed significant correlations with water content, MBP, MBN, NO₃^−^-N, inorganic-N, SOC, TN, AP, and pH. In contrast, the incomplete rTCA cycle displayed the most limited environmental associations, being significantly correlated only with tree richness and NO₃^−^-N.

Depth-stratified analysis revealed distinct environmental drivers of C fixation pathways between soil layers (Figure 4B). In topsoil, the Calvin cycle showed no significant correlations with measured factors, whereas the rTCA cycle correlated with MBN, NO₃^−^-N, and TN. The 3-HP cycle was linked to NO₃^−^-N, TN, and AP; the 3-HP/4-HB cycle only to AP. The Wood–Ljungdahl pathway correlated with MBC, NO₃^−^-N, and TN, and the incomplete rTCA cycle with NO₃^−^-N. In subsoil, the Calvin and rTCA cycles both correlated with tree density (and MBN for Calvin). The 3-HP cycle associated with tree density and TP, the Wood-Ljungdahl pathway with tree density, and the incomplete rTCA cycle with NO₃^−^-N. Overall, pathways were strongly influenced by nutrient availability (especially N and P), microbial biomass, and organic matter. These patterns indicate that topsoil pathways are primarily regulated by nutrient dynamics, while subsoil pathways respond more to tree community structure and microbial biomass.

### 3.4 Linkages of key function genes, metabolic pathways, and tree species diversity

**Figure 5:**
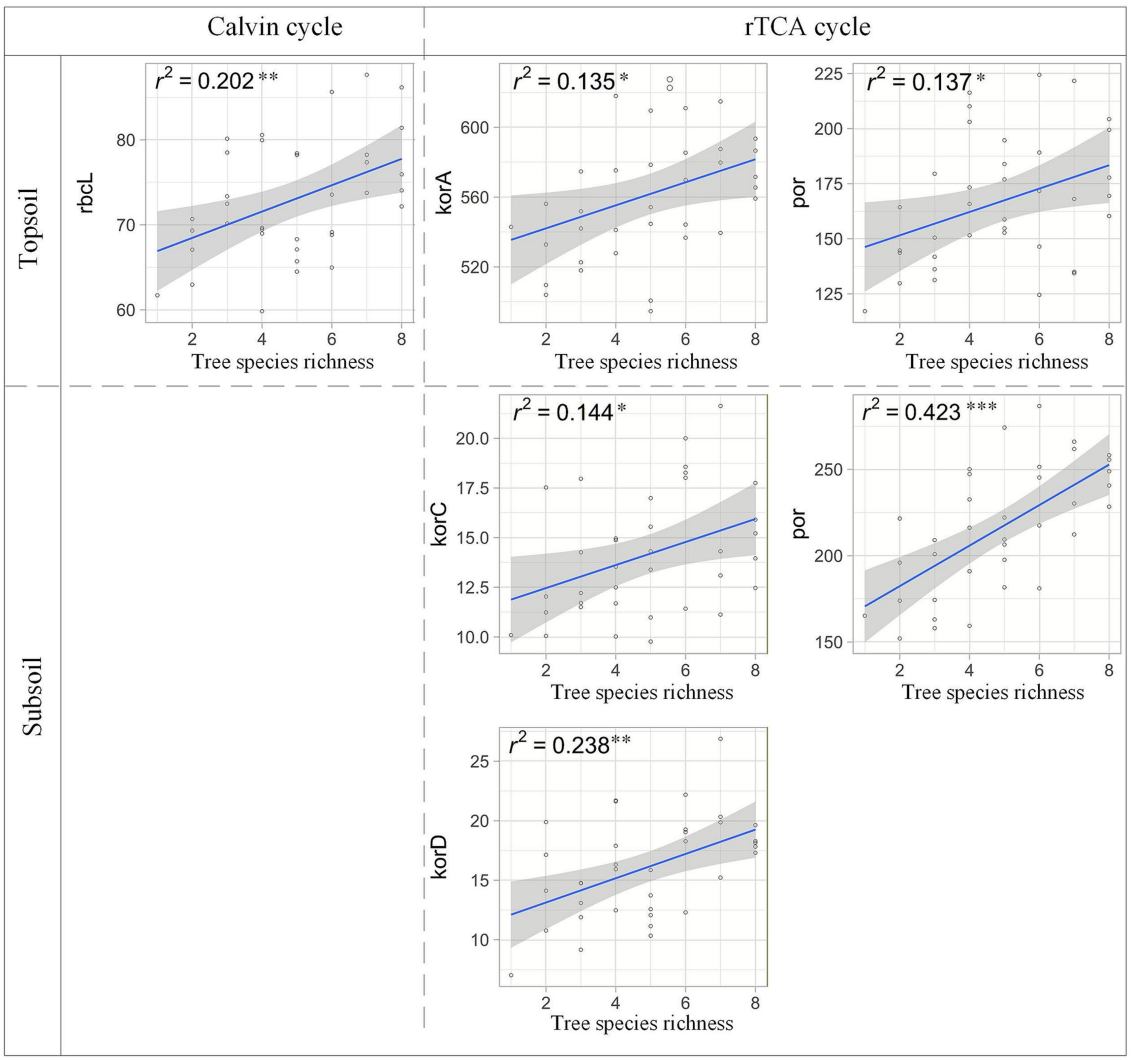
Relationships between tree species richness and microbial C fixation-related genes abundance. Solid lines are fit by ordinary least-squares regressions, and the shadow areas correspond to 95% confidence intervals. *p < 0.05, **p < 0.01, ***p < 0.001.

Linear regression revealed significant positive relationships between tree species richness and abundance of key functional genes from the rTCA and Calvin cycles (Figure 5). In topsoil, the abundance of *rbcL* (a key functional gene for the Calvin cycle) increased with tree species richness. In contrast, in subsoil, only the abundance of rTCA cycle-related genes (*korC, korD*, *por*) exhibited positive relationships with tree species richness.

**Figure 6:**
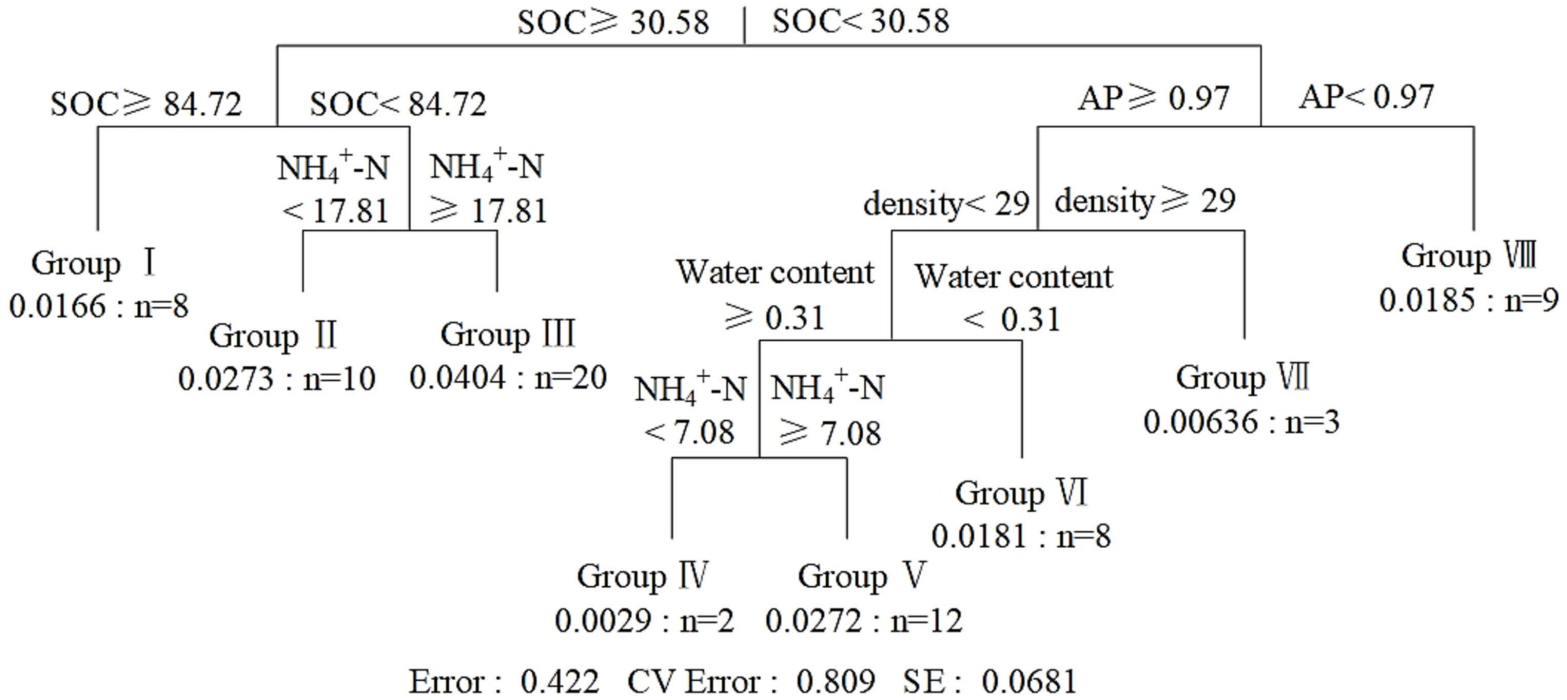
Results from multiple regression tree (MRT) for classification showing the indication of key genes for diverse clustering environments. SOC: Concentration of soil organic carbon (g kg^−1^); AP: soil available phosphorus (mg kg^−1^); NH_4_^+^-N: soil ammonium nitrogen (mg kg^−1^); density: tree density in each plot.

Multiple regression tree (MRT) analysis divided the dataset into eight subgroups based on soil factors (Figure 6). Samples were stratified into 8 groups based on 6 soil and stand factors, with threshold values for SOC (30.58 and 84.72 g kg^−1^), AP (0.97 mg kg^−1^), NH_4_^+^-N (7.08 and 17.81 mg kg^−1^), tree density (29 per 100 m^2^), and water content (0.31). Bar charts for each group (Supplementary Figure S2) showed the distribution of genes filtered by indicator value (*p* < 0.05) for each group (Table 2) identifying genes highly sensitive to environmental gradients, and suggesting their potential as habitat indicators.

**Table 2:** MRT cluster indicator gene filtering.

Group	Indicator	*P*-value	Indicator value	Metabolic pathways
I	*mct*	0.007	0.17	3-HP
	*E5.4.99.2B*	0.033	0.14	3-HP, 3-HP/4-HB
	*E2.2.1.1*	0.035	0.13	Calvin
	*pps*	0.005	0.16	rTCA, DC/4-HB
	*porC*	0.018	0.19	rTCA, DC/4-HB, Incomplete rTCA
	*fchA*	0.027	0.16	Wood-Ljungdahl
II	*GAPDH*	0.039	0.14	Calvin
III	*cdhD*	0.026	0.42	Wood-Ljungdahl
IV	*E5.4.99.2A*	0.009	0.14	3-HP, 3-HP/4-HB
	*glpX*	0.001	0.16	Calvin
	*K15016*	0.041	0.19	DC/4-HB, 3-HP/4-HB
	*frdA*	0.002	0.17	rTCA
	*ACO*	0.002	0.13	rTCA
	*ccsA*	0.034	0.19	rTCA
	*ccl*	0.007	0.27	rTCA
	*E4.2.1.2AA*	0.001	0.21	rTCA, DC/4-HB, Incomplete rTCA
	*sucD*	0.008	0.14	rTCA, DC/4-HB, Incomplete rTCA
	*pycA*	0.005	0.36	rTCA, Incomplete rTCA
	*pycB*	0.003	0.48	rTCA, Incomplete rTCA
V	*smtA1*	0.033	0.29	3-HP
VI	*E4.2.1.2B*	0.04	0.15	rTCA, 3-HP
	*sdhA*	0.037	0.14	rTCA, 3-HP, DC/4-HB
VII	*accB*	0.006	0.15	3-HP
	*K14469*	0.037	0.35	3-HP
	*meh*	0.005	0.17	3-HP
	*K15020*	0.005	0.34	3-HP/4-HB
	*FBA*	0.006	0.15	Calvin
	*glpX. SEBP*	0.048	0.14	Calvin
	*E4.2.1.2A*	0.044	0.14	rTCA
	*sdhD*	0.006	0.16	rTCA
	*sdhC*	0.008	0.15	rTCA, 3-HP, DC/4-HB
VII	*rpiB*	0.035	0.16	Calvin
	*PC*	0.043	0.15	rTCA
	*porD*	0.031	0.21	rTCA, DC/4-HB, Incomplete rTCA

The indicator genes with *p*-values greater than 0.05 were ignored.

## 4 Discussion

### 4.1 Vertical stratification of soil properties shapes depth-specific microbial C fixation strategies

Clear vertical stratification of soil physicochemical properties was observed in this forest ecosystem, with topsoil characterized by lower pH but higher SOC, nutrient availability, and microbial biomass than subsoil. Such depth-dependent gradients are widely reported across forest biomes and reflect long-term litter inputs, root distribution, and biogeochemical turnover processes (Qiao et al., 2020; Dai et al., 2023). Correspondingly, C fixation pathways exhibited pronounced depth-specific patterns, indicating that vertical heterogeneity in soil environments plays a key role in structuring microbial metabolic strategies. Rather than reflecting a simple dichotomy between aerobic and anaerobic metabolism, the observed partitioning of C fixation pathways along soil depth likely arises from microscale redox heterogeneity within soil matrices. Recent studies using redox-sensitive probes and microsensors have demonstrated that sharp oxygen gradients can occur over millimeter scales within soil aggregates, creating coexisting aerobic, microaerophilic, and anaerobic niches even within bulk-oxygenated topsoil layers (Sexstone et al., 1985; Keiluweit et al., 2020; Zhang and Furman, 2021). Such fine-scale redox heterogeneity provides diverse metabolic niches that can simultaneously support multiple C fixation strategies.

Within this context, the relative dominance of the Calvin cycle in topsoil may not simply reflect uniformly oxic conditions, but rather the combined effects of higher substrate availability, rapid carbon turnover, and metabolic flexibility. Although the Calvin cycle has relatively high ATP requirements, it can remain competitive in environments where energy and carbon substrates are abundant, allowing microorganisms to sustain energetically costly but versatile fixation pathways (Bar-Even et al., 2010; Hügler and Sievert, 2011). This interpretation is consistent with the weak environmental sensitivity of Calvin-cycle genes observed in topsoil, where resource availability may reduce selective pressure along single environmental gradients. In contrast, the enrichment of rTCA-related genes in subsoil likely reflects selection for energetically more efficient C fixation pathways under combined carbon, nutrient, and electron-acceptor limitation. The rTCA cycle requires lower ATP investment per unit of fixed carbon than the Calvin cycle and is therefore better suited to resource-constrained and microaerophilic environments (Berg et al., 2010; Bar-Even et al., 2010). Although we did not directly measure soil redox potential, the consistent decline in nutrient availability, microbial biomass, and organic matter with depth, together with well-established depth-dependent oxygen limitation in forest soils, supports this interpretation (Keiluweit et al., 2020; Naz et al., 2022). Overall, these findings highlight the importance of incorporating vertical redox and resource heterogeneity into assessments of microbial C fixation and forest carbon sequestration.

### 4.2 Tree species diversity enhances C fixation function through nutrient-mediated and functional gene regulation

Our GBM model revealed that tree species diversity and soil environmental factors jointly drive variation in C fixation gene abundance through significant nonlinear responses and interactive effects (Figure 3A). SOC and NO₃^−^-N were identified as the most important predictors, consistent with prior research emphasizing the role of soil organic matter and nitrogen. For example, in alpine meadows, SOC and C: N significantly explained variation in community abundance/structure (Duan et al., 2022). Long-term nitrogen deposition studies in temperate grasslands similarly demonstrated that NO₃^−^-N and soil N: P significantly affected the abundance of carbon-fixing bacteria (Fan et al., 2021). In restored ecosystems, research has indicated that SOC fractions and NO₃^−^-N are primary drivers of C fixation pathways/genes (Liang et al., 2024). Critically, our partial dependence analysis (Figure 3B) identified a threshold at ~85 g kg^−1^ SOC, beyond which the promotional effect on gene abundance declined or plateaued-a pattern not captured by conventional linear models. This nonlinear response-characterized by a sharp increase in gene abundance up to 80–85 g kg^−1^ SOC followed by saturation-suggests a physiological transition in microbial metabolic strategy as substrate availability shifts from limiting to non-limiting. One plausible mechanism involves feedback inhibition at the enzymatic level: as SOC concentration exceeds the capacity of microbial carbon-processing enzymes, metabolic intermediates may accumulate, suppressing further expression of C fixation genes, suggesting a shift in microbial energy allocation from growth to maintenance metabolism (Cui et al., 2024; Hu et al., 2025).

More fundamentally, the SOC threshold may reflect a trade-off between autotrophic and heterotrophic carbon acquisition strategies. When organic carbon is abundant, the energetic return on investment for directly metabolizing available substrates exceeds that of fixing atmospheric CO₂ via high-ATP pathways like the Calvin cycle. Our results—showing that Calvin-cycle gene (*rbcL*) abundance peaks at intermediate SOC and declines thereafter (Figure 5)—are consistent with this “catabolite repression” effect, whereby microorganisms downregulate costly autotrophic machinery when labile carbon is non-limiting (Cui et al., 2024). In contrast, rTCA-cycle genes (*korC, korD*) maintained positive relationships with tree richness even in high-SOC environments (Figure 5), suggesting that this energetically efficient pathway remains favored under nutrient-rich but electron-acceptor-limited conditions.

This study also revealed complex interactive effects among these factors. We found that C fixation gene abundance exhibited a nonlinear response pattern to SOC concentration, indicating a threshold effect of SOC on microbial C fixation function, which has rarely been explicitly quantified in previous research (Wang et al., 2017). Similar threshold effects in microbial carbon metabolism characteristics and environmental responses have been observed in other studies. For instance, Yu et al. (2025) found a threshold-type nonlinear relationship between microbial carbon use efficiency (CUE) and mean annual temperature. This threshold effect may reflect physiological constraints on microbial metabolism. Under high substrate concentration conditions, microorganisms may experience “metabolic congestion,” whereby excess substrate leads to metabolite accumulation, inhibiting key enzyme activities (Yin et al., 2024). Additionally, previous studies have suggested that microorganisms in high SOC environments may adopt a “low growth, high maintenance” survival strategy, allocating more energy to cell maintenance and repair rather than growth and functional gene expression, thereby reducing CUE (Cui et al., 2024). This shift in energy allocation caused by increased maintenance metabolism is considered one of the important mechanisms for microbial adaptation to carbon-rich environments (Hu et al., 2025; Shi et al., 2025).

The most novel finding of this study is the quantified interaction between tree richness and SOC (Figure 3C). Two-way partial dependence plots revealed that SOC’s promotional effect on gene abundance was strongest at intermediate tree richness (3–5 species), peaking near 80 g kg^−1^ SOC; however, as richness increased beyond 5 species, the SOC effect progressively weakened and plateaued. This pattern can be explained by the “Niche Saturation” and “Functional Redundancy” hypotheses (Loreau and Hector, 2001; Louca et al., 2018). In low-diversity stands, limited root exudate variety leaves microbial functional niches under-occupied, such that increases in SOC provide a strong stimulus by alleviating substrate limitation. However, as tree diversity increases, heterogeneous root architectures and diverse exudates enhance niche complementarity, effectively “filling” the available functional space (Sasse et al., 2018; Zhalnina et al., 2018). Beyond a threshold diversity level, our data suggest that the microbial community enters a functionally saturated state: additional species or higher SOC inputs no longer yield marginal gains in gene abundance, likely because the metabolic capacity of the community has reached a stoichiometric or physical limit.

This interaction demonstrates that tree diversity acts as a biotic filter determining the sensitivity of soil microbiomes to environmental shifts. In high-diversity plots, the microbial C fixation potential becomes more resilient but less responsive to further nutrient additions—our linear regression showed that the slope of the richness–gene abundance relationship declined when SOC exceeded 85 g kg-^1^ (Figure 5). These findings challenge the assumption that maximizing tree density or organic inputs will linearly enhance belowground carbon sequestration. Instead, they emphasize that optimizing species composition—particularly maintaining 3–5 functionally diverse species—can maximize the marginal return of soil carbon inputs on microbial autotrophic function. In the context of subtropical forest restoration and management, this suggests that biodiversity conservation not only preserves aboveground carbon stocks but also stabilizes belowground carbon cycling by buffering microbial communities against nutrient fluctuations.

## 5 Conclusion

Our study elucidates the relationships between tree species diversity and soil microbial C fixation at the metabolic pathway level in subtropical forests. We demonstrate that tree species diversity enhances soil microbial C fixation gene abundance, a process mediated by soil nutrients (particularly SOC and NO₃^−^-N) and stand density. Key functional genes of the Calvin and rTCA cycles showed positive but depth-dependent responses to tree species richness. SOC was the most important environmental factor, exhibiting a nonlinear threshold effect on gene abundance, while N and P availability also played critical roles. Consequently, the interaction between tree species diversity and soil properties drives distinct spatial strategies in microbial C fixation genes across soil layers. These findings highlight the importance of preserving tree diversity to optimize belowground carbon sequestration. Nevertheless, certain limitations should be noted: gene abundance served as a functional proxy, and direct activity measurements (e.g., metatranscriptomics or ^13^C tracing) would strengthen causal links. While depth-related pathway patterns align with oxygen-gradient theory, *in situ* redox data (e.g., Eh, O₂) are needed for mechanistic validation. Future studies should therefore integrate multi-omics and isotopic tracing with long-term monitoring across natural diversity gradients to better predict microbial carbon-fixation behavior under changing environments.

## Data Availability

The data supporting this study’s findings are available from the corresponding author upon reasonable request. And the raw sequence data reported in this paper have been deposited in the Genome Sequence Archive (Genomics, Proteomics and Bioinformatics 2021) in National Genomics Data Center (Nucleic Acids Res 2024), China National Center for Bioinformation/Beijing Institute of Genomics, Chinese Academy of Sciences (PRJCA055460) that are publicly accessible at https://ngdc.cncb.ac.cn/gsa.

## Footnotes

1
cloud.majorbio.com
