# Supplementary material for: Tree species diversity promotes soil microbial carbon fixation gene abundance via nutrient-mediated interactions in subtropical forests
Source: Front Microbiol. 2026 Feb 10;17:1751295. doi: 10.3389/fmicb.2026.1751295 (PMC12929538; doi:10.3389/fmicb.2026.1751295)
Supplement: Supplementary file 1 [file Table_1.DOCX]

Supplementary Materials

**Table S1.** Forest structure factors of the diameter at breast height (DBH, at 1.3 m above the ground), height , basal area , and stand density in the two 1.0-hectare permanent plots. PMF represents the forest dominated by *Pinus massoniana* and CGF represents the forest dominated by *Cyclobalanopsis glauca*.

|  | PMF | CGF |
| --- | --- | --- |
| Average DBH (cm) | 8.5 (4.0–30.8) | 9.1 (4.0–33.9) |
| Average height (m) | 12.7 (5.6–26.4) | 10.3 (6.4–24.2) |
| basal area (cm^2^ m^−2^) | 34.6 (18.6–56.4) | 32.6 (19.6–54.0) |
| Stand density (trees ha^−1^) | 6103 (2384–12480) | 5024 (1999–11120) |

**Table S2.** Plant community information of the subplots.

| Subplot | Species richness | Shannon-Wiener index | Evenness index | Tree density  （trees 100m^-2^） | Shrub species richness |
| --- | --- | --- | --- | --- | --- |
| 1 | 3 | 0.84 | 0.76 | 22 | 2 |
| 2 | 2 | 0.53 | 0.76 | 18 | 3 |
| 3 | 4 | 0.94 | 0.68 | 26 | 1 |
| 4 | 2 | 0.61 | 0.88 | 30 | 0 |
| 5 | 3 | 0.73 | 0.67 | 36 | 2 |
| 6 | 8 | 1.81 | 0.87 | 19 | 1 |
| 7 | 4 | 1.03 | 0.74 | 26 | 1 |
| 8 | 8 | 1.75 | 0.84 | 23 | 4 |
| 9 | 7 | 1.72 | 0.88 | 21 | 2 |
| 10 | 5 | 1.15 | 0.71 | 22 | 1 |
| 11 | 6 | 1.03 | 0.57 | 28 | 2 |
| 12 | 6 | 1.35 | 0.75 | 25 | 6 |
| 13 | 7 | 1.63 | 0.84 | 19 | 5 |
| 14 | 5 | 1.38 | 0.86 | 28 | 4 |
| 15 | 8 | 1.76 | 0.84 | 25 | 3 |
| 16 | 6 | 1.52 | 0.85 | 19 | 3 |
| 17 | 5 | 1.10 | 0.68 | 33 | 2 |
| 18 | 4 | 0.82 | 0.59 | 38 | 8 |
| 19 | 8 | 1.97 | 0.95 | 11 | 9 |
| 20 | 2 | 0.45 | 0.65 | 12 | 0 |
| 21 | 5 | 1.47 | 0.91 | 11 | 5 |
| 22 | 3 | 0.89 | 0.81 | 12 | 7 |
| 23 | 6 | 1.61 | 0.90 | 10 | 4 |
| 24 | 4 | 1.21 | 0.87 | 15 | 5 |
| 25 | 5 | 1.32 | 0.82 | 17 | 1 |
| 26 | 6 | 1.44 | 0.80 | 16 | 2 |
| 27 | 2 | 0.45 | 0.65 | 6 | 1 |
| 28 | 1 | 0 | 1 | 10 | 2 |
| 29 | 3 | 0.77 | 0.70 | 21 | 2 |
| 30 | 3 | 0.76 | 0.69 | 14 | 4 |
| 31 | 8 | 1.52 | 0.73 | 26 | 8 |
| 32 | 4 | 1.20 | 0.86 | 14 | 6 |
| 33 | 7 | 1.45 | 0.75 | 25 | 4 |
| 34 | 7 | 1.77 | 0.91 | 21 | 5 |
| 35 | 4 | 0.90 | 0.65 | 14 | 8 |
| 36 | 5 | 1.45 | 0.90 | 12 | 6 |

**Table S3** Primers of candidate genes for qPCR.

| Gene name | Primer sequence (5’ - 3’) | Size (bp) |
| --- | --- | --- |
| *rbcL* | TATCTTGGCAGCATTCCGAGTA  ACCCTCTTCAAATAGGTCTAATGG | 228 |
| *korA* | ATGACCGAACAGGTTATCCG  TCAGGCGTAACCGATGTAGT | 152 |

**Figure S1**

**
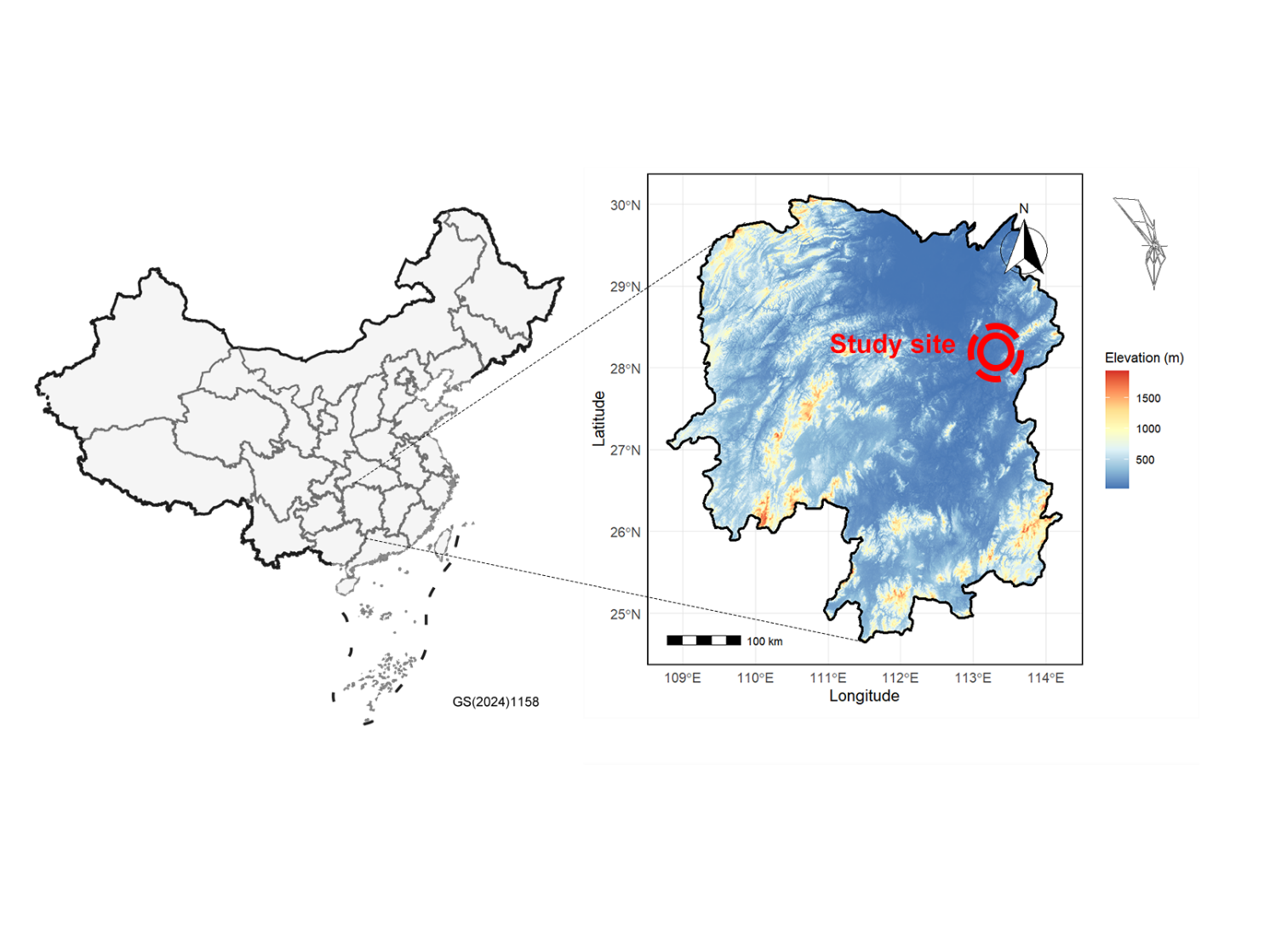
**

**Fig. S1** The study site is located in Dashanchong Forest Park, Changsha County, Hunan Province, China.

**Figure S2**

**
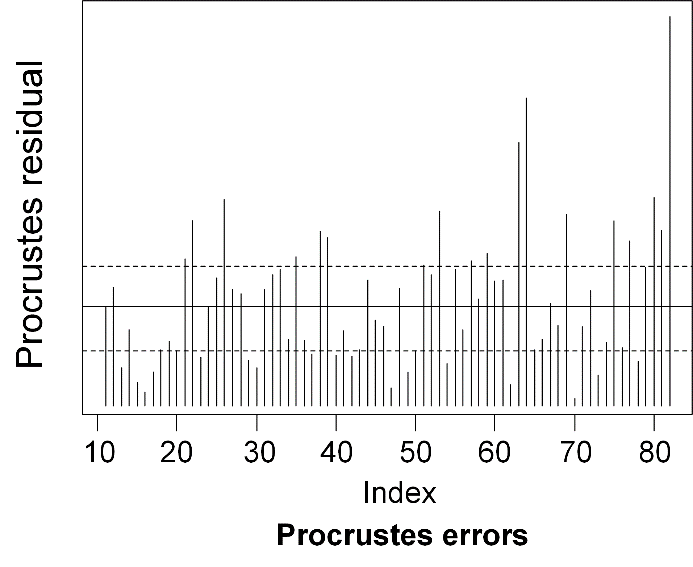
**

**Fig. S2** The figure shows the residuals for each set of paired values in the Procrustes analysis, with the horizontal lines from the bottom to the top being the 25% (dashed), 50% (solid), and 75% (dashed) quartiles of the residuals.

.

**Figure S3**


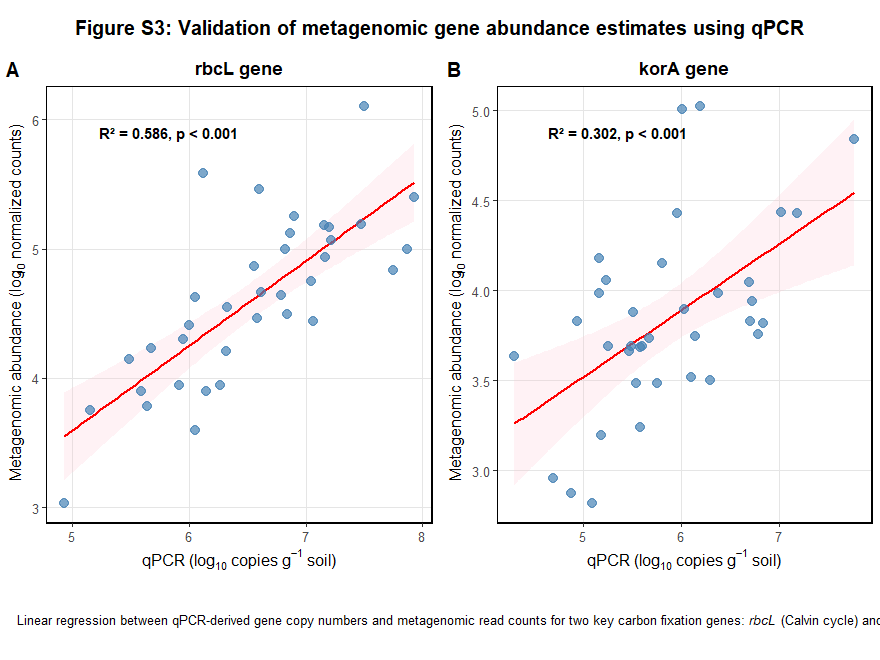


**Fig. S3** Linear regression between qPCR-derived gene expression levels and metagenomic sequencing abundance.

**Figure S4**


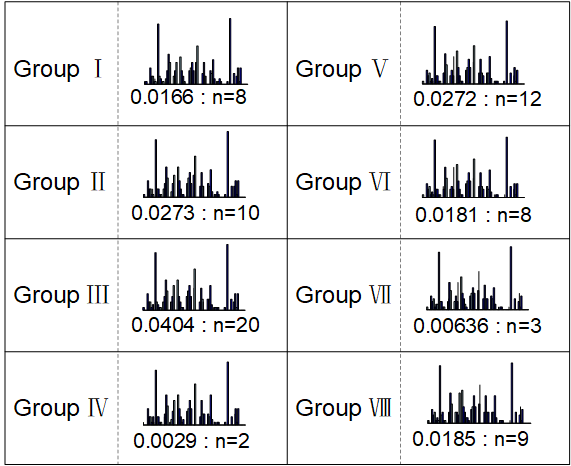


**Fig. S4** Bar chart of the contribution of samples from the gene samples in the multiple regression tree (MRT) analysis after the samples were divided into 8 groups according to the environmental factors. “n” represents the number of samples within the group.
